# Cardiac Inflammation in Adult-Onset Genetic Dilated Cardiomyopathy

**DOI:** 10.3390/jcm12123937

**Published:** 2023-06-09

**Authors:** Maurits A. Sikking, Sophie L. V. M. Stroeks, Michiel T. H. M. Henkens, Max F. G. H. M. Venner, Xiaofei Li, Stephane R. B. Heymans, Mark R. Hazebroek, Job A. J. Verdonschot

**Affiliations:** 1 Department of Cardiology, Maastricht University Medical Centre, Cardiovascular Research Institute Maastricht (CARIM), 6229 HX Maastricht, The Netherlands; m.sikking@maastrichtuniversity.nl (M.A.S.); s.stroeks@maastrichtuniversity.nl (S.L.V.M.S.); max.venner@maastrichtuniversity.nl (M.F.G.H.M.V.); s.heymans@maastrichtuniversity.nl (S.R.B.H.); mark.hazebroek@mumc.nl (M.R.H.); 2 Department of Pathology, Maastricht University Medical Centre, 6229 HX Maastricht, The Netherlands; michiel.henkens@mumc.nl (M.T.H.M.H.); xiaofei.li@mumc.nl (X.L.); 3 Netherlands Heart Institute (NLHI), 3511 EP Utrecht, The Netherlands; 4 Centre for Molecular and Vascular Biology, Department of Cardiovascular Sciences, Katholieke Universiteit Leuven, 3000 Leuven, Belgium; 5 Department of Clinical Genetics, Maastricht University Medical Centre, 6229 HX Maastricht, The Netherlands

**Keywords:** inflammation, genetics, dilated cardiomyopathy, diagnosis, myocarditis

## Abstract

Dilated cardiomyopathy (DCM) has a genetic cause in up to 40% of cases, with differences in disease penetrance and clinical presentation, due to different exogeneous triggers and implicated genes. Cardiac inflammation can be the consequence of an exogeneous trigger, subsequently unveiling a phenotype. The study aimed to determine cardiac inflammation in a cohort of genetic DCM patients and investigate whether it associated with a younger disease onset. The study included 113 DCM patients with a genetic etiology, of which 17 had cardiac inflammation as diagnosed in an endomyocardial biopsy. They had a significant increased cardiac infiltration of white blood, cytotoxic T, and T-helper cells (*p* < 0.05). Disease expression was at a younger age in those patients with cardiac inflammation, compared to those without inflammation (*p* = 0.015; 50 years (interquartile range (IQR) 42–53) versus 53 years (IQR 46–61). However, cardiac inflammation was not associated with a higher incidence of all-cause mortality, heart failure hospitalization, or life-threatening arrhythmias (hazard ratio 0.85 [0.35–2.07], *p* = 0.74). Cardiac inflammation is associated with an earlier disease onset in patients with genetic DCM. This might indicate that myocarditis is an exogeneous trigger unveiling a phenotype at a younger age in patients with a genetic susceptibility, or that cardiac inflammation resembles a ‘hot-phase’ of early-onset disease.

## 1. Introduction

Dilated cardiomyopathy (DCM) is a common non-ischemic cause of heart failure with an estimated 10-year survival rate free from death or heart transplantation of up to 85% [1]. A combination of environmental factors on top of a genetic background causes systolic dysfunction, increased afterload, and ventricle dilation in the absence of any abnormal loading condition or significant coronary artery disease [2]. Mutations within the genetic background were previously classified on their associated morphological and physiological phenotype (e.g., DCM, or hypertrophic cardiomyopathy). Since it is increasingly recognized that underlying genes overlap between cardiomyopathies, the view on which cardiomyopathies are classified shifted towards a genotype-phenotype approach in which genetic and molecular patterns define the etiology of cardiomyopathies [2,3,4,5].

Genetic DCM reflects up to 40% of the total DCM population [6,7]. It is characterized by left or biventricular dilation in the presence of a pathogenic or likely pathogenic (P/LP) variant in a gene robustly associated with DCM [4]. However, there is a major overlap between genetic DCM and DCM due to other etiologies [8]. As an example, there is an increased prevalence of truncating variants in *TTN* (TTNtv) in patients with DCM due to chemotherapy, alcohol abuse, or pregnancy [9,10,11]. Significant inflammation of the heart (i.e., myocarditis, defined by the most recent position statement of the European Society of Cardiology as a combined increase of leukocytes and T-cells in the myocardium [12]) is also a well-known etiology of DCM, and recent evidence showed an increased prevalence of genetic variants in acute and chronic myocarditis [13,14,15,16]. However, it remains unclear whether (1) cardiac inflammation is an environmental cause that unveils the penetrance of a P/LP variant at a younger age, or (2) if cardiac inflammation shares a pathophysiological overlap with genetic DCM in which dysfunctional peptides or chronic myocardial damage secondary to the genetic variant trigger a T-cell mediated immune response (i.e., myocarditis) as a consequence of the genetic variant [13,14]. For example, in a cohort of 107 patients with pathogenic variants in *DSP*, cardiac inflammation and fibrosis was detected in patients without systolic dysfunction, suggesting that inflammation and fibrosis may precede the development of systolic dysfunction in *DSP*-associated DCM [14]. Likewise, 1 in 13 (8%) of acute myocarditis patients had a P/LP variant in a population-based multinational analysis [13], suggesting that myocarditis often precedes the development of DCM in those with a genetic variant.

We hypothesize that cardiac inflammation is an independent predictor of young disease onset in patients with a P/LP variant in a DCM-associated gene.

## 2. Methods

### 2.1. Study Design and Population

We selected non-ischemic, nonvalvular DCM patients with a P/LP variant from the Maastricht Cardiomyopathy Registry from the Maastricht University Medical Center (MUMC) that prospectively includes individuals with heart failure-like symptoms or who underwent cardiac and/or genetic testing, enrolled between April 2004 and April 2021. The DCM diagnosis was defined according to the World Health Organization criteria [17] and the latest European Society of Cardiology (ESC) proposal [18]. The inclusion and exclusion criteria of the registry have been described previously [19]. For this study, ambulatory probands with DCM were included if they had: (1) a P/LP variant identified by genetic testing with our 48-cardiomyopathy-associated gene panel (Table A1) [8], and (2) undergone endomyocardial biopsy (EMB) as part of their diagnostic work-up. The study was performed according to the Declaration of Helsinki and was approved by the institutional Medical Ethics Committee. All patients gave written informed consent.

### 2.2. Endomyocardial Biopsy

Endomyocardial biopsies (EMB) were taken from the right ventricular septum via the internal jugular vein using a transcatheter bioptome (Cordis, Miami, FL, USA). Biopsies were collected as part of routine diagnostics for DCM to identify other triggers related to DCM, such as inflammation, storage diseases and metabolic disorders. Two specimens were used for immunohistological analysis on four µm-thick tissue sections from formalin-fixed, paraffin- embedded EMBs, and stained with hematoxylin and eosin, Sirius red, CD3^+^, CD45^+^, and CD68^+^, as part of routine clinical care. Cardiac inflammation was based on the latest position statement of the European Society of Cardiology (ESC) [12]: at least 14 leukocytes per mm^2^, including up to 4 macrophages, per mm^2^, and at least 7 T-lymphocytes per mm^2^.

### 2.3. Genetic Testing and Variant Classification

All patients received genetic testing using targeted next-generation sequencing (NGS) panels including 48 cardiomyopathy-associated genes (Table A1). A family history of cardiac-related disease and sudden cardiac death was obtained by pedigree analysis. Familial inheritance was defined as recommended by the ESC: (1) ≥2 individuals (first- or second-degree relatives) have DCM fulfilling diagnostic criteria for definite disease; or (2) in the presence of an index patient fulfilling diagnostic criteria for DCM and a first-degree relative with autopsy-proven DCM and sudden death at <50 years of age. All patients were also included in our previous study [20], in which we accurately curated and classified all detected variants according to the latest ACMG guidelines. All included patients had a pathogenic or likely pathogenic variant in one of the 48 cardiomyopathy-associated genes included in the panel.

### 2.4. Clinical Follow-Up and Outcomes

Follow-up data on mortality, heart failure hospitalization (HFH), and life-threatening arrhythmias (LTA), were collected using patients’ medical records, municipal population registers, and/or telephone contact with general practitioners. LTAs were defined as non-fatal ventricular fibrillation (with or without ICD-shock), hemodynamic unstable sustained ventricular tachycardia, and/or sustained ventricular tachycardia with appropriate ICD-shock. End of follow-up was defined by 31 September 2022. The median follow-up duration was 5.2 years. A combined endpoint of cardiac death, HFH, and LTA was used to determine event-free survival following the diagnosis of DCM.

### 2.5. Statistical Analysis

Penetrance was expressed as inversed Kaplan–Meier plots and compared between genetic DCM patients with and without myocarditis using multivariable Cox proportional-hazard regression analysis using the date of birth and the date of DCM diagnosis as baseline and primary endpoint, respectively. A *p*-value < 0.05 was considered statistically significant. All analyses were carried out using R (version 4.0.4).

## 3. Results

### 3.1. Study Population

In total, 113 patients with DCM and a P/LP variant were included in this study, of which 17 had cardiac inflammation in their EMB (15%). Overall, there were no significant differences between patients with or without inflammation, except for age (see Table 1). We observed a trend that patients with cardiac inflammation are more often female (47% versus 24%, *p*-value = 0.074) and have a higher left ventricle ejection fraction at DCM diagnosis (median 35% [IQR 23–48%] versus 30% [IQR 20–40%], *p*-value = 0.087). 6 (35%) genetic DCM patients with cardiac inflammation presented in the acute setting compared to 23 (24%) genetic DCM patients without cardiac inflammation (*p* = 0.370). All other patients presented in the outpatient setting. There were no significant differences between indication of EMB between the two study groups (*p* = 0.417; Table A2).

### 3.2. Histopathological Findings

**Figure 1:**
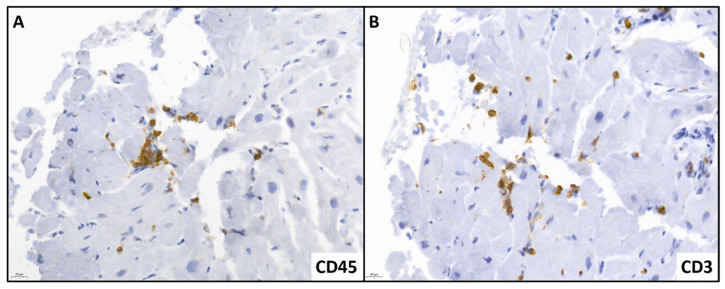
Representative sample of cardiac tissue with cardiac inflammation showing an increased cell count of CD45^+^ (**A**), and CD3^+^ (**B**) inflammatory cells in the myocardial tissue. The scale bars of the figures are depicted in the bottom left corner of the images, and display the length of 20 µm.

A detailed description of histological findings of genetic DCM patients with cardiac inflammation compared to genetic DCM patients without cardiac inflammation is provided in Table 2. There was a higher density of white blood cells, and helper and cytotoxic T-cells in the myocardium of genetic DCM patients with cardiac inflammation compared to those without cardiac inflammation (*p*-value < 0.001, <0.001, and <0.001 respectively). Additionally, there was a nonsignificant trend of a higher density of monocytes/macrophages, a higher collagen volume fraction, and a higher viral load (*p*-value 0.154, 0.186, and 0.402 respectively). There was no myocytolysis in any biopsy. A representative sample of cardiac tissue with cardiac inflammation is shown in Figure 1.

### 3.3. Pathogenic Variant Distribution between Groups

Pathogenic variants were equally distributed among genes between both study groups and were most prevalent in *TTN* (29% versus 49%, *p*-value = 0.19), followed by *LMNA* in (18% versus 9%, *p*-value = 0.39), and *MYBPC3* (12% versus 5%, *p*-value = 0.28; Table 3). All other genes harboring (likely) pathogenic variants were in the group without cardiac inflammation.

### 3.4. Penetrance Analysis

**Figure 2:**
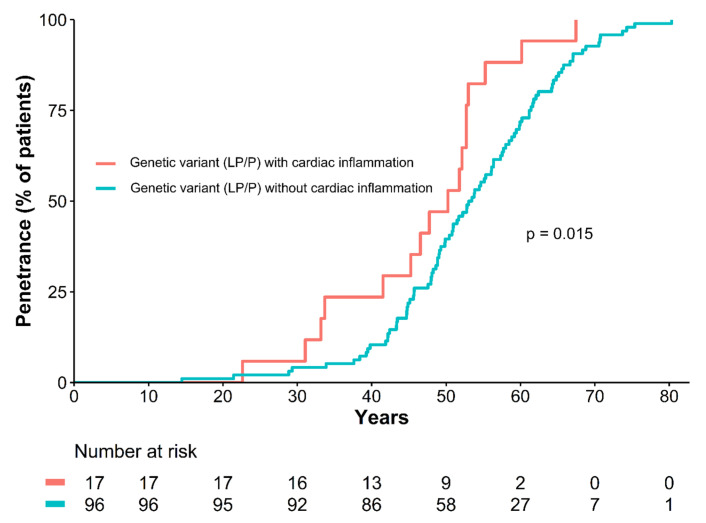
Cardiac inflammation associates with earlier penetrance of cardiomyopathy-associated genetic mutations. Penetrance is expressed as inversed Kaplan–Meier curves. Myocarditis is associated with an earlier disease onset in patients with genetic DCM independent of sex (HR 1.99, 95% confidence interval 1.14–3.45, *p* = 0.015).

Disease expression was at a younger age in those patients with a P/LP variant and cardiac inflammation, compared to those without inflammation (Figure 2; *p* = 0.015 after adjustment for sex). The median age of diagnosis for patients with a genetic variant and cardiac inflammation was 50 years [IQR 42–53] compared to 53 years [IQR 46–61] of those without cardiac inflammation (*p*-value = 0.034).

### 3.5. Clinical Outcome Analysis

**Figure 3:**
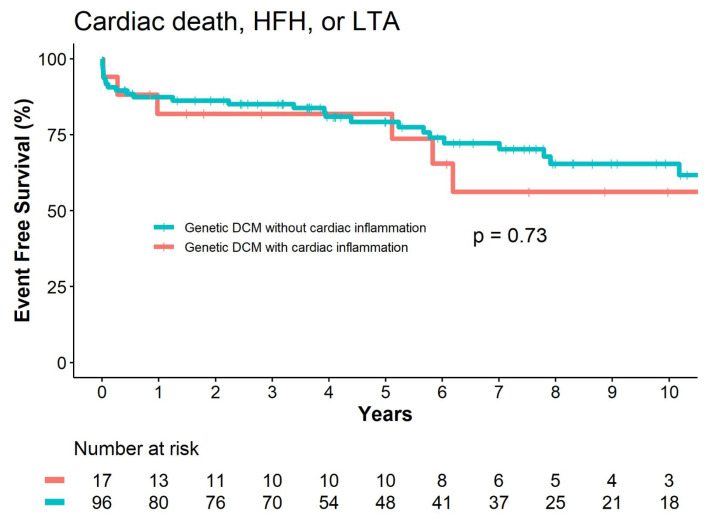
Event-free survival based on a combined endpoint of cardiac death, heart failure hospitalization, and life-threatening arrhythmia following genetic dilated cardiomyopathy diagnosis stratified by the presence or absence of significant cardiac inflammation.

There were no significant differences in long-term follow-up based on the combined endpoint of cardiac death, HFH, and LTA, comparing genetic DCM patients with presence to absence of cardiac inflammation (HR 0.85, 95%CI 0.35–2.07, *p* = 0.74, Figure 3). Likewise, there were no significant differences between each separate event type that constituted the combined endpoint: cardiac death (HR 1.27, 95%CI 0.28–5.67, *p* = 0.76), HFH (HR, 1.18 95%CI 0.27–5.24, *p* = 0.83), and LTA (HR, 0.49 95%CI 0.16–1.54, *p* = 0.22).

**Figure A1:**
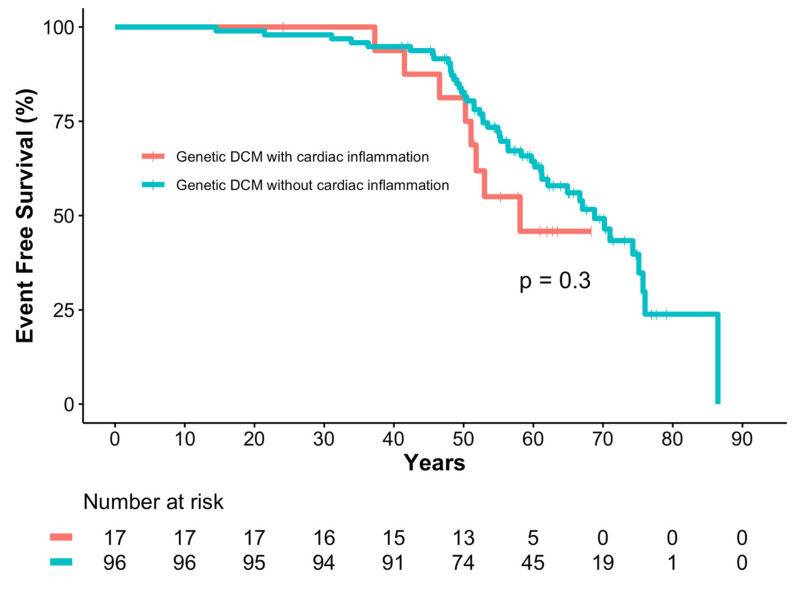
Event-free survival based on a combined endpoint of cardiac death, heart failure hospitalization, and life-threatening arrhythmia of genetic dilated cardiomyopathy patients from birth stratified by the presence or absence of significant cardiac inflammation.

There were not significant differences in the combined endpoint of cardiac death HFH, and LTA comparing genetic DCM patients with presence to absence of cardiac inflammation if date of birth was taking as the baseline of the analysis (HR 0.60, 95%CI 0.27–1.30, *p* = 0.19, Figure A1).

## 4. Discussion

In this cross-sectional study of patients with DCM and a P/LP variant, we observed that 15% had significant cardiac inflammation in their cardiac biopsy, and that the presence of cardiac inflammation is associated with a younger disease onset but not with a worse outcome.

### 4.1. Association between Genetics, Cardiac Inflammation, and Myocarditis

We previously showed that 18% of patients with DCM and cardiac inflammation had a P/LP variant in a DCM-associated gene, which is comparable to patients with idiopathic DCM [8]. In addition, previous studies showed enrichment of pathogenic variants in patients with acute myocarditis [13,14]. Pathogenic variants were identified in 8% of myocarditis cases compared to <1% of healthy controls, suggesting that a trigger for myocarditis unveils an earlier phenotype in those with a P/LP variant, or that myocarditis shares a pathophysiological overlap with genetic cardiomyopathy [13]. One could argue that in the presence of a P/LP variant, the heart is more prone to evolve towards DCM after myocarditis. Our results show that significant cardiac inflammation, which reflects a chronic form of myocarditis, is associated with a younger disease onset. This emphasizes the earlier studies in which genetic testing in patients with myocarditis is recommended, as it has the potential to stratify patients who are more likely to develop a DCM. In addition, the finding of a P/LP enables the possibility of identifying family members who are at increased risk of developing DCM.

We observed cardiac inflammation as defined by the latest position statement of the European Society of Cardiology. However, the clinical implication of the detected inflammation remains unknown, as the cause of the inflammation remains unknown. Inflammation can be the consequence of an exogenous trigger, such as a viral infection. We performed analysis of the most prevalent cardiotropic viruses in cardiac tissue but did not find a significant enrichment in those patients with cardiac inflammation. In addition, the presence of cardiac inflammation was not associated with a worse outcome in patients with genetic DCM.

### 4.2. Inflammation as Consequence of P/LP Variants in Genes

We did not find a significant association between specific genes and cardiac inflammation, i.e., there was no enrichment of a gene in the group of patients with DCM and cardiac inflammation. Desmoplakin (*DSP*) is, by far, most associated with inflammation among all the DCM-associated genes, and episodes of myocardial inflammation associated with *DSP* cardiomyopathy might be confused with (viral) cardiomyopathy [14,21]. DSP links other desmosomal proteins (desmocollin-2 and desmoglein-2), that are by themselves anchored in the plasma membrane, to desmin, which is a structural protein anchoring multiple structures (e.g., Z-disc, mitochondria) within the cytoplasm [22,23]. Cardiac specific overexpression of desmocollin-2 led to acute inflammation and biventricular cardiomyopathy in a transgenic mouse model [23]. The cardiac inflammation is most often diagnosed by observing myocardial edema on cardiac magnetic resonance imaging (MRI) that may suggest an inflammatory process [21,24], and is present together with episodes of chest pain associated with elevated cardiac enzymes [25]. Myocardial inflammation has become a key feature of arrhythmogenic cardiomyopathy, mainly in the young, where the inflammation might resemble a ‘hot-phase’ of the disease [26]. This ‘hot-phase’ might be a feature of the initial disease manifestation, but may also might be an important contributor to disease progression and arrhythmogenesis, wherein overlap between myocarditis and arrhythmogenic cardiomyopathy may be assumed [27]. We did not find a P/LP variant in *DSP* in our cohort that could have contributed to the observed cardiac inflammation, and we detected P/LP variants in prevalent DCM-associated genes.

Belkaya et al. found that silent recessive defects in cardiomyopathy-associated genes predisposes the myocardium to heart failure presenting as acute myocarditis, notably after common viral infections in children [28]. We did not detect any homozygous or compound heterozygous variant in any of our patients, although we only included adult patients. In addition, Hata et al. described that unexplained minimal inflammatory foci in the absence of structural heart disease encountered during autopsy might be an early sign of inherited cardiomyopathy [29], emphasizing the importance of inflammation in the disease development of cardiomyopathy due to a P/LP variant.

### 4.3. Study Strengths, Limitations, and Future Research Opportunities

A significant strength of our study is the availability of cardiac tissue in a relatively large cohort of genetic DCM patients, resembling a unique cohort. With the increasing association between cardiac inflammation and genetic cardiomyopathy, our study was able to perform an association analysis in an unselected cohort of patients with DCM.

The main limitation of this study is the retrospective single-center design. We performed a cross-sectional study, therefore we do not know the causality between cardiac inflammation and the DCM phenotype in our patient cohort. We only observed that patients with a P/LP variant and cardiac inflammation are overall younger compared to those without cardiac inflammation. Moreover, according to the guidelines, we only take endomyocardial biopsies when the left ventricle ejection fraction remains decreased despite optimal medical therapy. This could lead to an underestimation of cardiac inflammation in our population, as those with a recovered ejection fraction still could have significant cardiac inflammation.

Future studies should focus on the follow-up of myocarditis patients with and without a P/LP variant to analyze clinical parameters that can predict DCM development. This is an unmet need in risk assessment of young individuals with myocarditis, in which genetic testing could be of value. Our study underlines the value of genetic counseling in DCM patients with cardiac inflammation. Together with factors such as biventricular dysfunction, fulminant presentation, this could determine the follow-up protocol of myocarditis patients. Likewise, studies on family members of genetic DCM patients may integrate inflammatory parameters to predict (early) onset of disease.

## 5. Conclusions

Cardiac inflammation is prevalent in patients with genetic forms of DCM and is associated with an earlier disease onset. This might indicate that myocarditis is an exogeneous trigger unveiling a phenotype at a younger age in patients with a genetic susceptibility, or that cardiac inflammation resembles a ‘hot-phase’ of early-onset disease. Future studies should aim to unravel the consecutive development of inflammation and overt disease development in patients with genetic DCM.

## Figures and Tables

**Table 1:** Demographics of all included patients in the current study at the time of genetic DCM diagnosis.

	Genetic DCM with Cardiac Inflammation (*n* = 17)	Genetic DCM without Cardiac Inflammation (*n* = 96)	*p*-Value
Age, years	50 (42–53)	53 (46–61)	0.034
Female sex	8 (47.1%)	23 (24.0%)	0.074
Atrial fibrillation	3 (17.6%)	17 (17.7%)	1.000
Diabetes mellitus type 2	1 (5.9%)	6 (6.2%)	1.000
Arterial hypertension	2 (11.8%)	26 (27.1%)	0.233
Asthma	2 (11.8%)	8 (8.3%)	0.645
COPD	1 (5.9%)	6 (6.2%)	1.000
Previous cancer	1 (5.9%)	5 (5.2%)	1.000
NYHA ≥ III	1 (5.9%)	12 (12.5%)	0.707
LVEF, %	35 (23–48)	30 (20–40)	0.087

Values represent median and interquartile range (IQR), or number and percentage.

**Table 2:** Description of measurements in cardiac biopsies of patients with DCM, including individual cell counts, viral presence, and viral load.

	Genetic DCM with Cardiac Inflammation (*n* = 17)	Genetic DCM without Cardiac Inflammation (*n* = 96)	*p*-Value
White blood cells, per mm^2^	13.3 [11.6–16.9]	5.8 [3.9–7.8]	<0.001
T-cells, per mm^2^	9.7 [8.0–12.7]	3.6 [2.0–5.5]	<0.001
Helper T-cells, per mm^2^	5.1 [4.2–7.3]	1.7 [1.0–2.8]	<0.001
Cytotoxic T-cells, per mm^2^	6.9 [3.5–7.9]	1.9 [1.0–3.0]	<0.001
Monocytes/macrophages, per mm^2^	3.8 [2.2–4.7]	1.5 [0.8–3.1]	0.154
Collagen volume fraction in %	11.5 [5.0–20.6]	6.3 [3.6–13.4]	0.186
Significant viral load *	5 (29.4%)	21 (22.6%)	0.544

Values represent median and interquartile range (IQR), or number and percentage. * Significant viral load was defined as at least 200 copies per microgram DNA.

**Table 3:** Distribution of pathogenic or likely pathogenic variants in DCM-associated genes among patients with DCM and with or without cardiac inflammation.

	Genetic DCM with Cardiac Inflammation (*n* = 17)	Genetic DCM without Cardiac Inflammation (*n* = 96)	*p*-Value
*TTN*	5 (29)	47 (49)	0.19
*LMNA*	3 (18)	9 (9)	0.39
*MYBPC3*	2 (12)	5 (5)	0.28
*MYH7*	1 (6)	6 (6)	0.99
*NEXN*	1 (6)	0 (0)	0.15
*RBM20*	1 (6)	3 (3)	0.48
*SCN5A*	1 (6)	0 (0)	0.15
*TNNC1*	1 (6)	0 (0)	0.15
*TNNT2*	2 (12)	2 (2)	0.11

Values represent number and percentage.

## Data Availability

Data is available upon reasonable request.

## Appendix A

**Table A1:** Overview of all 48 genes used in the Maastricht Cardiomyopathy gene-panel.

	HGNC.ID	HGNC.Symbol	HGNC.Name
1	HGNC:143	*ACTC1*	Actin, alpha, cardiac muscle 1
2	HGNC:164	*ACTN2*	Actinin alpha 2
3	HGNC:15819	*ANKRD1*	Ankyrin repeat domain 1
4	HGNC:939	*BAG3*	BCL2 associated athanogene 3
5	HGNC:20407	*CALR3*	Calreticulin 3
6	HGNC:1529	*CAV3*	Caveolin 3
7	HGNC:2389	*CRYAB*	Crystallin alpha B
8	HGNC:2472	*CSRP3*	Cysteine and glycine rich protein 3
9	HGNC:2511	*CTNNA3*	Catenin alpha 3
10	HGNC:2770	*DES*	Desmin
11	HGNC:3036	*DSC2*	Desmocollin 2
12	HGNC:3049	*DSG2*	Desmoglein 2
13	HGNC:3052	*DSP*	Desmoplakin
14	HGNC:3331	*EMD*	Emerin
15	HGNC:3702	*FHL1*	Four and a half LIM domains 1
16	HGNC:4296	*GLA*	Galactosidase alpha
17	HGNC:14202	*JPH2*	Junctophilin 2
18	HGNC:6207	*JUP*	Junction plakoglobin
19	HGNC:6484	*LAMA4*	Laminin subunit alpha 4
20	HGNC:6501	*LAMP2*	Lysosomal associated membrane protein 2
21	HGNC:15710	*LDB3*	LIM domain binding 3
22	HGNC:6636	*LMNA*	Lamin A/C
23	HGNC:21086	*MIB1*	Mindbomb E3 ubiquitin protein ligase 1
24	HGNC:7551	*MYBPC3*	Myosin binding protein C, cardiac
25	HGNC:7576	*MYH6*	Myosin heavy chain 6
26	HGNC:7577	*MYH7*	Myosin heavy chain 7
27	HGNC:7583	*MYL2*	Myosin light chain 2
28	HGNC:7584	*MYL3*	Myosin light chain 3
29	HGNC:1330	*MYOZ2*	Myozenin 2
30	HGNC:23246	*MYPN*	Myopalladin
31	HGNC:29557	*NEXN*	Nexilin F-actin binding protein
32	HGNC:9024	*PKP2*	Plakophilin 2
33	HGNC:9080	*PLN*	Phospholamban
34	HGNC:14000	*PRDM16*	PR/SET domain 16
35	HGNC:9386	*PRKAG2*	Protein kinase AMP-activated non-catalytic subunit gamma 2
36	HGNC:27424	*RBM20*	RNA binding motif protein 20
37	HGNC:10593	*SCN5A*	Sodium voltage-gated channel alpha subunit 5
38	HGNC:11577	*TAZ*	Tafazzin
39	HGNC:11610	*TCAP*	Titin-cap
40	HGNC:28472	*TMEM43*	Transmembrane protein 43
41	HGNC:11943	*TNNC1*	Troponin C1, slow skeletal and cardiac type
42	HGNC:11947	*TNNI3*	Troponin I3, cardiac type
43	HGNC:11949	*TNNT2*	Troponin T2, cardiac type
44	HGNC:12010	*TPM1*	Tropomyosin 1
45	HGNC:12403	*TTN*	Titin
46	HGNC:12405	*TTR*	Transthyretin
47	HGNC:12665	*VCL*	Vinculin
48 *	HGNC:3756	*FLNC*	Filamin C

* FLNC was only sequenced in 172/689 (25%) DCM patients, since the gene was added to the panel in June 2018.

**Table A2:** Indication of endomyocardial biopsy according to the 2021 Position statement on endomyocardial biopsy from the Heart Failure Association of the European Society of Cardiology, Heart Failure Society of America, and Japanese Heart Failure Society.

	Genetic DCM with Cardiac Inflammation (*n* = 17)	Genetic DCM without Cardiac Inflammation (*n* = 96)	*p*-Value
EMB indication			0.417
Autoimmune disorders with progressive HF unresponsive to treatment with/without sustained ventricular arrhythmias and/or conduction abnormalities	2 (11.8%)	12 (12.5%)	
Suspected fulminant myocarditis or acute myocarditis with acute HF, LV dysfunction and/or rhythm disorders	2 (11.8%)	4 (4.2%)	
DCM with recent onset HF, moderate-to-severe LV dysfunction, refractory to standard treatment (following exclusion of specific etiologies)	13 (76.5%)	80 (83.3%)	

Values represent number and percentage.
